# Identification of neurodevelopmental transition patterns from infancy to early childhood and risk factors predicting descending transition

**DOI:** 10.1038/s41598-022-08827-4

**Published:** 2022-03-21

**Authors:** Takeo Kato, Tomoko Nishimura, Nagahide Takahashi, Taeko Harada, Akemi Okumura, Toshiki Iwabuchi, Yoko Nomura, Atsushi Senju, Kenji J. Tsuchiya, Nori Takei

**Affiliations:** 1 United Graduate School of Child Development, Hamamatsu University School of Medicine, Hamamatsu, 431-3192 Japan; 2 Research Center for Child Mental Development, Hamamatsu University School of Medicine, Hamamatsu, 431-3192 Japan; 3 Department of Child and Adolescent Psychiatry, Nagoya University Graduate School of Medicine, Nagoya, 464-8601 Japan; 4 Queens College and Graduate Center, City University of New York, New York, NY 10031 USA; 5 Institute of Psychiatry, King’s College London, London, WC2R 2LS UK

**Keywords:** Epidemiology, Risk factors, Neurodevelopmental disorders

## Abstract

It is unclear whether neurodevelopmental progress from infancy to early childhood remains stable. Moreover, little is known about the risk factors, if any, affecting neurodevelopmental descending transition patterns and the relationship between these patterns and later childhood adaptive behaviours. We used data of 875 children from the Hamamatsu Birth Cohort Study in Japan. Children’s neurodevelopment at 18 and 32 months and adaptive behaviours at 40 months were evaluated. Perinatal factors and infant overweight status at 18 months were investigated to identify descending-transition-associated risk factors. In the latent transition analysis, ultimately, three classes were identified for each time-point, resulting in nine transition patterns; among them, 10.4% of children showed descending class shifts (normal to delayed class). Such decelerated growth was predicted by maternal pre-pregnancy overweight status (odds ratio [OR] 2.49; 95% confidence interval [CI] 1.23, 5.02), low maternal educational history (OR 1.20; 95% CI 1.04, 1.36), and infant overweight status at 18 months (OR 5.89; 95% CI 1.26, 27.45). Children with descending transition showed poor functioning in adaptive behaviours at the age of 40 months. To prevent subsequent poor adaptive functioning, it may be necessary to consider that a certain percentage of children show decelerated growth.

## Introduction

An understanding of the neurodevelopmental trajectories in early life provides a clue to predict developmental pathways in later life^1,2^. Neurodevelopmental delays during early infancy negatively impact childhood development^3^. Moreover, neurodevelopmental progress from infancy to early childhood may be inconsistent, and early delays develop in very heterogeneous ways^4^. Some children show typical development at first time-point of measurement but deviate from typical progress at a later time-point. However, these children do not necessarily exhibit developmental regression, which is defined as loss of previously acquired skills^5^. Such a developmental course, which we call a descending transition, has seldom been reported. On the other hand, catching-up transitions, referring to neurological maturation retardation at certain time-points^4^, are well-known. However, the term ‘developmental delays’ has no clear definition and is often arbitrarily defined using descriptive-statistical measures such as standard deviations and percentile values^6,7^. Therefore, it is critically important to define the neurodevelopmental status or subtype based on the achievement level of development at each time-point to comprehend the children’s neurodevelopmental transition. While many studies have comprehensively assessed early neurodevelopment and its trajectories^8,9^, no previous study has comprehensively evaluated a range of transition patterns which include the descending transition.

Furthermore, little is known about risk factors that may influence such descending transitions. Hillemeier et al.^6^ found that prenatal and obstetric factors such as low maternal education, low family income, and preterm birth had an increasing effect on descending transitions. However, the developmental pathways of children are also affected by postnatal factors, including physical growth, as well as biological and environmental factors^10^.

The relationship between a descending course of neurodevelopment during early childhood and untoward consequences in everyday functioning, including adaptive behaviours, at a later stage has attracted attention. While this connection has already been investigated, it only exists in a high-risk sample of siblings of children with autism spectrum disorder (ASD)^11^.

Hence, this study has three aims. First, in a birth cohort sample of Japanese children (18–32 months old), to apply a statistical modelling technique (latent transition analysis; LTA)^12^ to identify classes with distinct transition patterns. Second, to explore risk factors, including pre-pregnancy maternal variables, environmental indicators, and infant physical growth variables, which could predispose children to specific transition patterns, especially focusing on a diversion from normal to delayed development. Third, to determine whether children with descending transition patterns show poorer performance in adaptive behaviours at a subsequent stage (age 40 months).

## Methods

### Ethics approval

The study protocol was approved by the Hamamatsu University School of Medicine and University Hospital Ethics Committee (Ref Nos. 17-037 and 17-037-3) and performed in accordance with the Declaration of Helsinki. Written informed consent was obtained from each mother for her own and her infant’s participation.

### Participants

This study is part of the ongoing cohort study, the Hamamatsu Birth Cohort Study for Mothers and Children (HBC Study). Participants included mothers (*n* = 1138) and their infants (*n* = 1258) born between 24 December 2007 and 19 March 2012. The detailed recruitment procedure has been previously described^9^. The participants were determined to be a representative sample of the general Japanese population^13^.

In applying the exclusion criteria, the final sample in this study comprised 875 infants and 795 mothers (Supplementary Fig. S1); Two infants whose mothers had passed away before their first birthdays, an infant with a birth weight less than 1000 g, two infants diagnosed with Down syndrome, and twins and other multiple births (*n* = 25) were also excluded. Additionally, 353 infants who failed to participate in outcome assessments at 18 or 32 months of age (henceforth referred to as time 1 and time 2, respectively), or both, were excluded.

### Measures

#### Neurodevelopment

Neurodevelopmental progress was assessed using the Mullen Scales of Early Learning (MSEL) at time 1 and time 2^14^. MSEL is a validated composite scale for ascertaining child neurodevelopment through direct testing and is composed of five domains: gross and fine motor, visual reception, and expressive and receptive language. A Japanese version of the associated T-score, with a mean of 50 and a standard deviation (SD) of 10, was used as the outcome. The detail of the T-score and procedures of the direct testing of MSEL and retaining the reliability of assessments among clinical evaluators have been described previously^9^.

#### Risk factors for neurodevelopmental transition

Risk factors associated with neurodevelopmental transition patterns in children, especially descending patterns from time 1 to time 2, were explored (Supplementary Note 1). A perinatal factor, small-for-gestational-age (birth weight < 10th percentile for gestational age)^15^ was applied. Moreover, infant overweight status at time 1 was incorporated as another risk index^16^. Child weight and height at 18 months of age were obtained from records taken from periodic health check-ups. Child BMI values were transformed into a standardized body mass index (sBMI) corresponding to a BMI-for-age value with a mean of 0 and an SD of 1, based on the World Health Organization’s Child Growth Standards^17^. Subsequently, sBMI scores were dichotomised into ‘overweight’ (over 1 SD) and ‘others’. The mother’s pre-pregnancy overweight status was also included as another biological variable; self-reported pre-pregnancy BMI (kg/m^2^) during early pregnancy was dichotomised as ‘overweight’ (over 25 kg/m^2^) and ‘others’^18^. A history of maternal education (a continuous variable) obtained through an interview was also used.

##### Covariates

Infant sex, premature birth (< 37 weeks), low placenta-to-birth-weight ratio (< 10th percentile)^19^, parental age, paternal educational history, and annual household income were used as covariates for multinomial logistic regression. Data relating to the demographic characteristics of parents were collected during pregnancy, and perinatal variables were obtained from medical records.

#### Adaptive behaviours

The everyday functional levels of each child were quantified using the vineland adaptive behaviour scales second edition (VABS-II) at 40 months of age. The VABS is based on a semi-structured parental interview consisting of four domains: communication, daily living, socialization, and motor skills^20^. An overall adaptive behaviour composite standardized score, with a mean of 100 and an SD of 15, was used.

### Statistical analysis

#### Latent transition analysis

LTA was used to compute the latent transition probabilities which suggest the likelihood of individuals changing classes or remaining in the same class across consecutive periods^12^. LTA may compute transition probabilities at multiple time-points, such as three time-points; however, the precision of estimation might become inferior owing to the involvement of a multiplicative increase in parameters. Therefore, we opted for two time-points (e.g., time 1 and time 2) to achieve our goal, which was to identify classes with distinct transition patterns. Prior to the main analysis, five domains of MSEL T-scores at time 1 and time 2 were set as the outcome measures through latent class analysis (LCA). To select the appropriate class solution in latent class analysis (LCA), we used five common fit indices^21^: the Akaike information criterion (AIC), Bayesian information criterion (BIC), entropy, adjusted Lo-Mendell Rubin likelihood ratio test (LMR-LRT), and bootstrap likelihood ratio test (BLRT). Along with these indices, the number of classes was ultimately determined by considering theoretical justification and interpretability^22^. After the best solution at each time-point was identified via LCA, the transition probability by which each individual was assigned to an optimal transition pattern was computed using LTA without potential risk factors and covariates. For the LTA procedure, a slightly modified version of the three-step approach was followed (Supplementary Note 1)^23,24^. In this approach, potential risk factors (maternal pre-pregnancy overweight status, low maternal educational history, small-for-gestational-age, and infant overweight status at time 1) and covariates (infant sex, premature birth, placenta-to-birth-weight ratio, and household income) which differed across classes at time 1 were included in the analysis (Supplementary Note 1) and examined to determine which factors would emerge as affecting transition patterns (i.e., class shifts) using multinomial logistic regression.

#### Transition pattern effect on adaptive behaviours

The associations of transition patterns between time 1 and time 2, especially descending transitions, with adaptive behaviours at 40 months were investigated using linear regression analysis, wherein risk factors identified in LTA were included as covariates to account for their potential confounding effects. Familial clustering was controlled for using the Huber–Sandwich method. The statistical analyses in the present study were conducted using Mplus version 8 (https://www.statmodel.com/)^25^ and Stata version 14.0 (https://www.stata.com/stata14/)^26^.

##### Data attrition

Infants who received MSEL evaluations at time 1 and 2 were included. The rate of missing data from the five MSEL domains was minimal (1.6% in total). The full information maximum likelihood (FIML) method, a powerful tool for missing data, was used^27^. Any biases arising from the assumption of missing at random for FIML were therefore considered negligible.

## Results

### Neurodevelopmental transition patterns from infancy to early childhood

**Figure 1:**
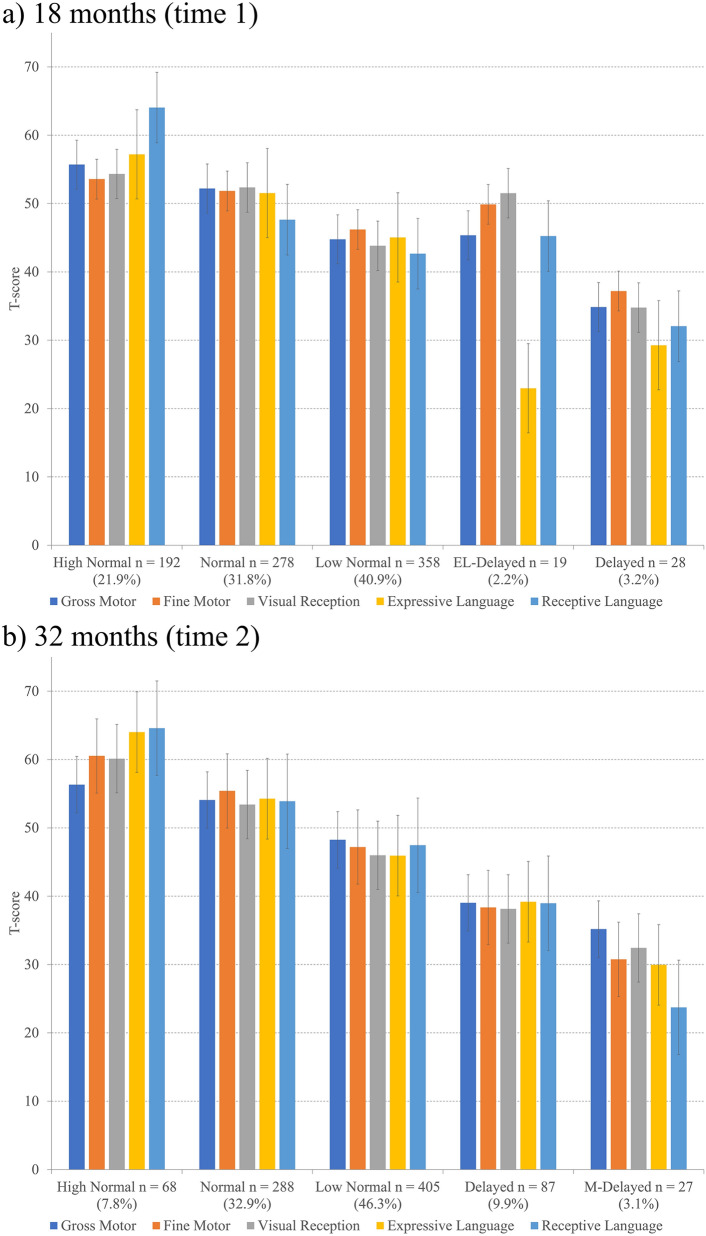
Latent class structure by neurodevelopmental domains at 18 months (**A**) and 32 months (**B**) (*n* = 875). *EL-delayed* expressive language delayed, *M-delayed* markedly delayed.

Table 1 shows the participants’ demographic characteristics included in the analysis. In LCA, fit indices in latent class analysis are shown in Supplementary Table S1. We investigated up to seven class solutions. For LCA time 1 (i.e., 18 months), the adjusted LMR-LRT showed *P* < 0.05 up to the five-class solution, whereas BIC was smallest for the six-class solution. AIC continuously reduced, while BLRT remained significant up to the seven-class solution. As for LCA time 2 (i.e., 32 months), the adjusted LMR-LRT similarly showed *P* < 0.05 up to the five-class solution, while BIC had the smallest value for the six-class solution. AIC continued to decrease, and BLRT showed *P* < 0.001 up to the seven-class solution. These findings indicate the optimal solution was five classes or more for both time 1 and time 2. In our previous study of the same birth cohort, we found that five classes would best delineate the distinctive trajectories of neurodevelopment over the period of infancy (1–24 months), including the current time-point (i.e., time 1)^9^. For comparisons and facilitation of interpretations, we opted for the five-class solution for both time 1 and time 2. Figure 1 shows the MSEL composition of the five classes identified by the LCA procedures. At time 1 (Fig. 1a), three classes within 50 ± 1 SD (i.e., leftmost to the third column in the figure) were designated as ‘normal’, consisting of ‘high normal’ (21.9%), ‘normal’ (31.8%), and ‘low normal’ (40.9%), in order from left to right. The fourth group of children (2.2%) showed a downward deviation (− 2 SD) only in the expressive language domain (denoted as ‘expressive language (EL)-delayed’). The last group (3.2%) showed low scores (below − 1 SD) in all five domains (‘delayed’). Similarly, three normal classes, ‘high normal’ (7.8%), ‘normal’ (32.9%), and ‘low normal’ (46.3%), were identified at time 2 (Fig. 1b). The fourth group (9.9%) showed scores below − 1 SD in all five domains (‘delayed’). The last group (3.1%) showed markedly low scores in all five domains (‘markedly (M)-delayed’).

**Table 1:** Characteristics of participating infants and their parents.

Infants	Mean (SD)
Birth weight (g)	2957.0 (422.2)
Gestational age at birth (weeks)	39.0 (1.4)

	n (%)
**Sex**	
Male	432 (49.4%)
Female	443 (50.6%)
**Prematurity**	
< 37 weeks	46 (5.3%)
≥ 37 weeks	829 (94.7%)
**Small-for-gestational-age**	
< 10th percentile	77 (8.8%)
10th–100th percentile	798 (91.2%)
**Age-standardized BMI scores at 18 months**	
> 1 standard deviation	50 (6.1%)
< 1 standard deviation	776 (93.9%)

Parents	n (%)
**Maternal BMI at pre-pregnancy (kg/m**^**2**^**)**	
> 25.0	100 (11.4%)
< 25.0	775 (88.6%)
**Placenta-to birth-weight ratio**	
< 10th percentile	152 (17.7%)
10th–100th percentile	707 (82.3%)

	Mean (SD)
Paternal age at birth (year)	33.5 (5.7)
Maternal age at birth (year)	31.7 (5.0)
Paternal education (year)	14.1 (2.6)
Maternal education (year)	13.8 (1.8)
Household income (million JPY)	6.0 (2.7)

*SD* standard deviation, *BMI* body mass index, *JPY* Japanese Yen.

Table 2 shows counts and proportions for each combination of class assignments from time 1 to time 2. Only a small number of children (*n* < 9, corresponding to less than 1%) was allocated to 12 out of the 25 observable transition classes, including the descending pattern classes. Thus, the three normal classes were amalgamated at each time-point and labelled as ‘3-normals’ to increase statistical power during relative risk estimations of children assigned to descending transition classes in comparison with baseline control classes (i.e., normal-to-normal transition patterns). Thus, the number of transition patterns was reduced to nine. Of the 875 infants included in the analyses, 737 (84.2%) were assigned to the reference transition pattern of ‘3-normals to 3-normals’ at both time-points, 91 (10.4%) to the descending transition patterns (“3-normals to delayed” and “3-normals to M-delayed”), while 24 (2.8%) were assigned to the catching-up transition patterns (“EL-delayed to 3-normals” and “delayed to 3-normals”), and 23 (2.6%) were assigned to the four transition patterns of remaining in delayed classes at both time-points.

**Table 2:** Transition class counts and proportions derived by latent transition analysis (*n* = 875).

			Latent class at time 2, n (%)				
			Combined 3-normals			Delayed 87 (9.9%)	M-delayed 27 (3.1%)
			High-Normal 68 (7.8%)	Normal 288 (32.9%)	Low-normal 405 (46.3%)		
Latent class at time 1, n (%)	Combined 3-normals	High-normal 192 (21.9%)	49 (5.6%)	87 (9.9%)	56 (6.4%)	0 (0%)	0 (0%)
		Normal 278 (31.8%)	12 (1.4%)	132 (15.1%)	130 (14.5%)	0 (0%)	4 (0.5%)
		Low-normal 358 (40.9%)	7 (0.8%)	61 (7.0%)	203 (23.2%)	74 (8.5%)	13 (1.5%)
		EL-delayed 19 (2.2%)	0 (0%)	6 (0.7%)	9 (1.0%)	3 (0.3%)	1 (0.1%)
		Delayed 28 (3.2%)	0 (0%)	2 (0.2%)	7 (0.8%)	10 (1.1%)	9 (1.0%)

*EL-delayed* expressive language delayed, *M-delayed* markedly delayed.

### Risk factors for transition patterns

Table 3 shows associations between the risk factors for infants with descending and catching-up transition patterns compared with infants with the reference transition pattern (i.e., “3-normals to 3-normals” at both time-points). Children whose mothers had a pre-pregnancy overweight status had a 2.49-fold increase in the risk of being assigned to the “3-normals to delayed” transition pattern than children with the reference transition pattern (odds ratio [OR] 2.49; 95% confidence interval [CI] 1.23, 5.02). Low maternal educational history was also associated with this descending transition pattern. A 1-year decrease in maternal educational history corresponded to a 20% increase in the risk of infants being assigned to the “3-normals to delayed” transition pattern (OR 1.20; 95% CI 1.04, 1.36). Overweight status at time 1 in infants was associated with being assigned to the “3-normals to M-delayed” transition pattern (OR 5.89; 95% CI 1.26, 27.45). For catching-up transition patterns, low maternal educational history was also associated with assignment to EL-delayed to 3-normals” transition pattern (OR 1.21; 95% CI 1.01, 1.49), and infants with SGA were more likely to be allocated to the “delayed to 3-normals” transition pattern (OR 10.13; 95% CI 2.23, 45.85). Associations between covariates and neurodevelopmental transition patterns in the final model are shown in Supplementary Table S2 and Note 2.

**Table 3:** Association between possible risk factors and neurodevelopmental transition patterns in the final model of multinomial logistic regression analysis with covariates (*n* = 811).

	Transition from 3-normals at time 1 (765)		
	To 3-normals at time 2 (681)	Delayed at time 2 (70)	Markedly delayed at time 2 (14)
	Reference	OR [95% CI]	OR [95% CI]
**Possible risk factors**			
Maternal overweight status at pre-pregnancy	··	2.49 [1.23, 5.02]*	1.27 [0.20, 8.02]
Low maternal education (year)	··	1.20 [1.04, 1.36]*	1.12 [0.74, 1.69]
Small-for-gestational-age	··	1.81 [0.72, 4.53]	0.89 [0.10, 7.93]
Infant overweight status at 18 months	··	1.49 [0.57, 3.88]	5.89 [1.26, 27.45]*

	Transition from expressive language delayed at time 1 (19)		
	To 3-normals at time 2 (15)	Delayed at time 2 (3)	Markedly delayed at time 2 (1)
	OR [95% CI]	OR [95% CI]	OR [95% CI]
**Possible risk factors**			
Maternal overweight status at pre-pregnancy	0.83 [0.10, 6.69]	NA	NA
Low maternal education (year)	1.21 [1.01, 1.49]*	0.91 [0.46, 1.81]	1.96 [1.58, 2.43]****
Small for gestational age	0.93 [0.11, 7.53]	3.85 [0.010, 1409.03]	NA
Infant overweight status at 18 months	NA	NA	NA

	Transition from delayed at time 1 (27)		
	To 3-normals at time 2 (9)	Delayed at time 2 (10)	Markedly delayed at time 2 (8)
	OR [95%CI]	OR [95%CI]	OR [95%CI]
**Possible risk factors**			
Maternal overweight status at pre-pregnancy	4.79 [0.91, 25.26]	0.90 [0.09, 8.57]	1.40 [0.17, 11.37]
Low maternal education (year)	1.09 [0.74, 1.61]	1.26 [0.85, 1.88]	1.42 [0.86, 2.32]
Small for gestational age	10.13 [2.23, 45.85]***	NA	NA
Infant overweight status at 18 months	2.78 [0.24, 31.99]	2.15 [0.24, 19.18]	NA

Infant’s sex (male), premature birth (< 37 weeks), low placenta-to-birthweight ratio (< 10th percentile), and household income.

Fifty infants who had missing values for infant’s sBMI at 18 months and 14 mother-infant dyads who had missing values for mother’s placental weight were excluded from this analysis.

*OR* odds ratio, *CI* confidence interval, *NA* not available.

*P < 0.05, ***P < 0.005, ****P < 0.001.

### Transition patterns and adaptive behaviour

Table 4 presents the associations between the nine transition patterns and the adaptive behaviour composite standardized scores at 40 months of age. Compared with children with the reference transition pattern, children with the two descending transition patterns showed lower adaptive scores 8 months later (“3-normals to delayed”: coefficient =  − 7.88; 95% CI − 9.77, − 6.00 and “3-normals to m-delayed”: coefficient =  − 13.04; 95% CI − 18.87, − 7.21). Children who stayed in delayed transition classes also showed poorer levels of adaptive ability. Conversely, children who achieved the two catching-up transition patterns had no significant differences in adaptive functioning when compared with infants in the reference transition group (“EL-delayed to 3-normals”: coefficient =  − 2.26; 95% CI − 5.04, 0.50 and “delayed to 3-normals”: coefficient = 0.50; 95% CI − 3.98, 4.99).

**Table 4:** Association between neurodevelopmental transition patterns and adaptive behaviour at 40 months in linear regression analyses with covariates (*n* = 779).

	Adaptive behaviour composite standardized score
	Coefficient [95% CI]
**Transition patterns (*n*)**	
3-Normals to 3-normals (654)	Reference
3-Normals to delayed (67)	− 7.88 [− 9.77, − 6.00]****
3-Normals to markedly delayed (13)	− 13.04 [− 18.87, − 7.21]****
EL-delayed to 3-normals (14)	− 2.26 [− 5.04, 0.50]
Delayed to 3-normals (9)	0.50 [− 3.98, 4.99]
EL-delayed to delayed (3)	− 11.49 [− 16.9, − 6.01]****
EL-delayed to markedly delayed (1)	− 14.13 [− 15.20, − 13.06]****
Delayed to delayed (10)	− 9.76 [− 13.39, − 6.13]****
Delayed to markedly delayed (8)	− 17.48 [− 21.77, − 13.19]****

*CI* confidence intervals, *EL-delayed* expressive language delayed.

****P < 0.001.

^a^Infant sex (male), premature birth (< 37 weeks), low placenta-to-birth-weight ratio (< 10th percentile), household income, maternal body mass index at pre-pregnancy (> 25 kg/m^2^), small-for-gestational-age (< 10th percentile), infant’s standardised body mass index at 18 months of age (> 1 SD), and maternal educational history.

### Data attrition

Notably, characteristics (i.e., sex, birth weight, gestational age at birth, and prematurity) of infants and parental background characteristics (i.e., parental age and maternal educational history) differed between the infants included and those excluded from the analyses (Supplementary Table S3). These differences may have biased the findings. The inverse probability weighting method^28^ was applied to the final model to allow for missing variations associated with these six variables. The estimates derived from the inverse probability weighting model for descending transition patterns remained statistically significant, with ORs of 2.2 (95% CI 1.32, 3.79) for maternal overweight at pre-pregnancy, 3.89 (95% CI 1.25, 12.06) for infant overweight at time 1, 4.16 (95% CI 1.40, 12.28) except for maternal educational history, which had an almost identical estimation but fell just short of statistical significance (OR 1.12; 95% CI 0.99, 1.26).

## Discussion

LTA was applied to multiple neurodevelopmental measures collected from infancy to early childhood in a Japanese birth cohort sample and found that 10.4% of children were diverted from normal neurodevelopment levels (3-normals) to lower levels, implying decelerated developmental patterns. Maternal pre-pregnancy overweight status, infant overweight status at time 1 (i.e., 18 months), and low maternal educational history were identified as risk factors associated with descending transition patterns. Further, children assigned to descending transition patterns or who remained in delayed neurodevelopmental classes from time 1 to time 2 (i.e., 32 months) demonstrated poorer adaptive functioning at 40 months. In contrast, children with catching-up transition patterns attained a normal range of adaptive functioning at 40 months.

Our study showed that 5.4% of infants (47/875) were assigned to the delayed classes at time 1 and 13.0% (114/875) to the delayed classes at time 2, indicating that there was an overall increase in the rate of delayed infants over the study period. Further, 10.4% of infants were identified as having descending transition patterns from time 1 to time 2. A previous study based on a Taiwanese nationwide sample^29^ reported that the prevalence of neurodevelopmental delay from 1 to 3 years of age increased about three to four-fold, suggesting that, akin to our findings, a substantial proportion of children show a descending developmental pattern from infancy to early childhood. Approximately half (51%) the infants (n = 24) who belonged to delayed classes (i.e., ‘expressive language,’ EL-delayed, and ‘delayed’ classes) at time 1 (n = 47) moved to normal classes (i.e., ‘3-normals’) at time 2, showing catching-up transition patterns. This finding is inconsistent with those of previous studies reporting that approximately 80–90% of children who initially belonged to a delayed class eventually caught up^6,7^. In these studies, however, ‘delayed class’ was arbitrarily defined using deviations from an average (e.g., below − 1 SD); thus, the prevalence of developmental delays in infancy might be overestimated. Infants in the EL-delayed class in our study may include the so-called ‘late talkers’ (LT), or children under 3 years old who have unusually small vocabularies with unknown primary causes^30^. Overall, children with LT have a good prognosis, with 60–70% of children moving into the average range on language measures by preschool^31^. Our study supports this finding in that 79% of infants assigned to the EL-delayed class at time 1 caught up to the normal classes by time 2.

Maternal pre-pregnancy overweight status was a risk factor for a descending transition pattern. This finding supports previous studies which associated maternal pre-pregnancy overweight status with motor^32^, language^33^, and cognitive neurocognitive developmental delay in offspring^34^. Maternal pre-pregnancy overweight status might produce a chronic systemic inflammatory ambience in both mother and foetus, with long-lasting negative consequences for neuronal development in offspring^35^ by affecting insulin and leptin levels and other inflammatory markers^36^. Girchenko et al.^37^ indicated that higher levels of maternal inflammation of high-sensitivity C-reactive protein and glycoprotein acetyls mediated the effect of prenatal environmental adversity including maternal early pregnancy overweight on child neurodevelopmental delay.

Infant overweight status at time 1 was considered another risk factor for descending transition patterns. Overweight status in infancy is known to be related to neurodevelopmental delays^16,38^ and neuroimaging research has accumulated evidence on the relationship between overweight status and structural changes in the brain^38,39^. Obesity may cause reductions in brain volume through inflammatory responses produced by adipose tissue^40^. Thus, overweight status during infancy, when brains are developing rapidly^41^, may negatively impact healthy brain development and lead to descending transition patterns in early childhood.

Low maternal education was associated with the descending transition pattern. Maternal educational history appears to be associated with variables indicating poor parenting environments, including low maternal responsivity and lack of effective communication^42^, and may result in more developmental differences^43^. Genetic or inherent influences may become more salient during the later stage of infancy. For example, a declining trajectory of development has frequently been reported in children with neurodevelopmental disorders such as ASD^44^, and lower levels of maternal education may be a risk factor for neurodevelopmental disorders^45^.

The results of the present study showed that the risk factors are independently associated with the descending neurodevelopmental trajectory. However, it has been reported that these risk factors are associated with each other. It has been reported that maternal obesity had an adverse effect on child obesity^46^, and obesity in early childhood was more commonly seen among those born to mothers with low education^47^. Therefore, further investigation is needed to determine the association among these risk factors and child neurodevelopmental trajectories including mediation and moderation.

Small for gestational age (SGA) was found to be positively associated with the catching-up transition pattern from the Delayed class to the 3-normals class. Although SGA infants reportedly have lower levels of cognitive, language, and motor development than infants with appropriate gestational age^48^, a catching-up phase of neurodevelopmental progress during early childhood has been noted in these children^49,50^, which is compatible with the findings of the present study. Growth hormone/insulin-like growth factors have been proposed to be possible mechanisms behind this catching-up phase in children with SGA^51^. Low maternal education level was also associated with catching-up transition patterns from the EL-delayed class to the 3-normals class. However, when the inverse probability weighting method was applied to the model, this finding was no longer significant (OR 1.17, 95% CI 0.91, 1.49), implying that the seeming association might reflect a bias arising from sample selection procedures.

There was no association between risk factors and persistent delayed transition patterns (i.e., from the EL-delayed class and the delayed class at time 1 to the delayed class and the markedly delayed, M-delayed, class at time 2), except that maternal education was associated with the transition pattern of EL-delayed to M-delayed. Although this exception was grounded on a small sample size (n = 1) and needs further investigations, it has been reported that there was an association between low maternal education and neurodevelopmental delay in children^43^.

It was found that infants with descending transition patterns and persistent delays showed lower adaptive behaviour scores at 40 months of age than infants with the reference transition pattern. Our findings regarding infants with persistent delays are consistent with a previous study, which identified an association between lower neurodevelopmental scores in infancy and poorer adaptive behaviours in siblings of children with ASD^11^. To the best of our knowledge, this is the first study to demonstrate the relationship between developmental transition patterns and later adaptive behaviour in a general population sample.

One of the main strengths of this study was that our population comprised a representative sample of infants; thus, our findings are generalizable. Second, this study examined five domains of neurodevelopment using established instruments and direct evaluations. Third, to the best of our knowledge, this study is the first to report risk factors for predicting descending transition patterns and subsequent poorer adaptive functioning in children at the early developmental stage.

However, this study has some limitations. First, the sample size was comparatively small. Among the 25 initially identified transition patterns, 12 were each composed of less than 1% of children; thus, the three normal classes were combined into a single “3-normals” at both time-points to avoid false-negative findings due to a lack of statistical power. Therefore, the findings, especially the risk factors, should be interpreted with caution. In fact, maternal BMI and infant sBMI were dichotomized in the present study, but when they were treated as continuous variables, the association with descending transition pattern was changed. Although the association between maternal BMI and the descending transition pattern remained significant (OR 1.08, 95% CI 1.01, 1.17), the infant sBMI did not (OR 2.10, 95% CI 0.80, 5.48). In future studies, we recommend verifying our findings through large sample sizes for possible application in clinical practice. Second, potential postnatal risk factors for predicting transition patterns were limited only to infant BMI. Further research is warranted to determine the impact of other growth-related indices, including protective factors, for infants, such as nutritional intake, interpersonal activities at early life stages, and environmental factors (i.e., living conditions and accessibility and utilization of health and social care services). Reliance on parental reports of the adaptive performance in their offspring might also have impacted the outcome measures. It is plausible that the performance of adaptive skills in children reported by parents may have been exaggerated^52^. Nevertheless, no evidence indicated that this bias occurred in combination with specific transition patterns of neurodevelopment; thereby, it is unlikely to have confounded the findings of the association between transition classes and adaptive functioning. However, there remains the possibility that our findings may be overrepresented by a subpopulation of infants, particularly, those with developmental impairments such as ASD. Acquired knowledge and information provided regarding the disorder may have influenced parents’ reporting attitudes in favour of the research questions in the present study. The results, however, remained virtually unchanged when children diagnosed with ASD at time 2 (*n* = 26) by paediatricians blinded to any research hypotheses were eliminated from the analyses (Supplementary Tables S4, S5), suggesting that our findings cannot be attributed to a specific group of developmental conditions.

In conclusion, this study underscored the dynamic nature of comprehensive neurodevelopmental progress from 18 to 32 months and identified risk factors associated with descending neurodevelopmental transition patterns during this period. In this representative sample of Japanese children, 10.4% showed descending transition patterns, which were predicted by maternal pre-pregnancy overweight status, low maternal educational history, and infant overweight status at 18 months. It was also found that children with descending transition patterns had lower adaptive behaviours at 40 months. Further studies are warranted to replicate our findings using independent samples in large studies.

## Supplementary Information

Supplementary Information.

## Data Availability

The datasets used and/or analysed during the current study are available from the corresponding author on reasonable request.
