# Supplementary material for: Identification of neurodevelopmental transition patterns from infancy to early childhood and risk factors predicting descending transition
Source: Sci Rep. 2022 Mar 21;12:4822. doi: 10.1038/s41598-022-08827-4 (PMC8938496; doi:10.1038/s41598-022-08827-4)
Supplement: Supplementary file 1 — Supplementary Information. [file 41598_2022_8827_MOESM1_ESM.pdf]

Online supplementary information for the "Identification of neurodevelopmental transition patterns from infancy to early childhood, and risk factors predicting descending transition

Takeo Kato,[1,2] Tomoko Nishimura,[1,2] Nagahide Takahashi,[2,3] Taeko Harada,[1,2] Akemi Okumura,[1,2] Toshiki Iwabuchi,[1,2] Yoko Nomura,[2,4] Atsushi Senju,[1,2] Kenji J. Tsuchiya,[1,2] and Nori Takei[1,2,5]

1. United Graduate School of Child Development, Hamamatsu University School of Medicine, Hamamatsu, 431-3192, Japan;
2. Research Center for Child Mental Development, Hamamatsu University School of Medicine, Hamamatsu, 431-3192, Japan;
3. Department of Child and Adolescent Psychiatry, Nagoya University Graduate School of Medicine, Nagoya, 464-8601, Japan;
4. Queens College and Graduate Center, City University of New York, NY, 10031, USA;
5. Institute of Psychiatry, King's College London, WC2R 2LS, London, UK

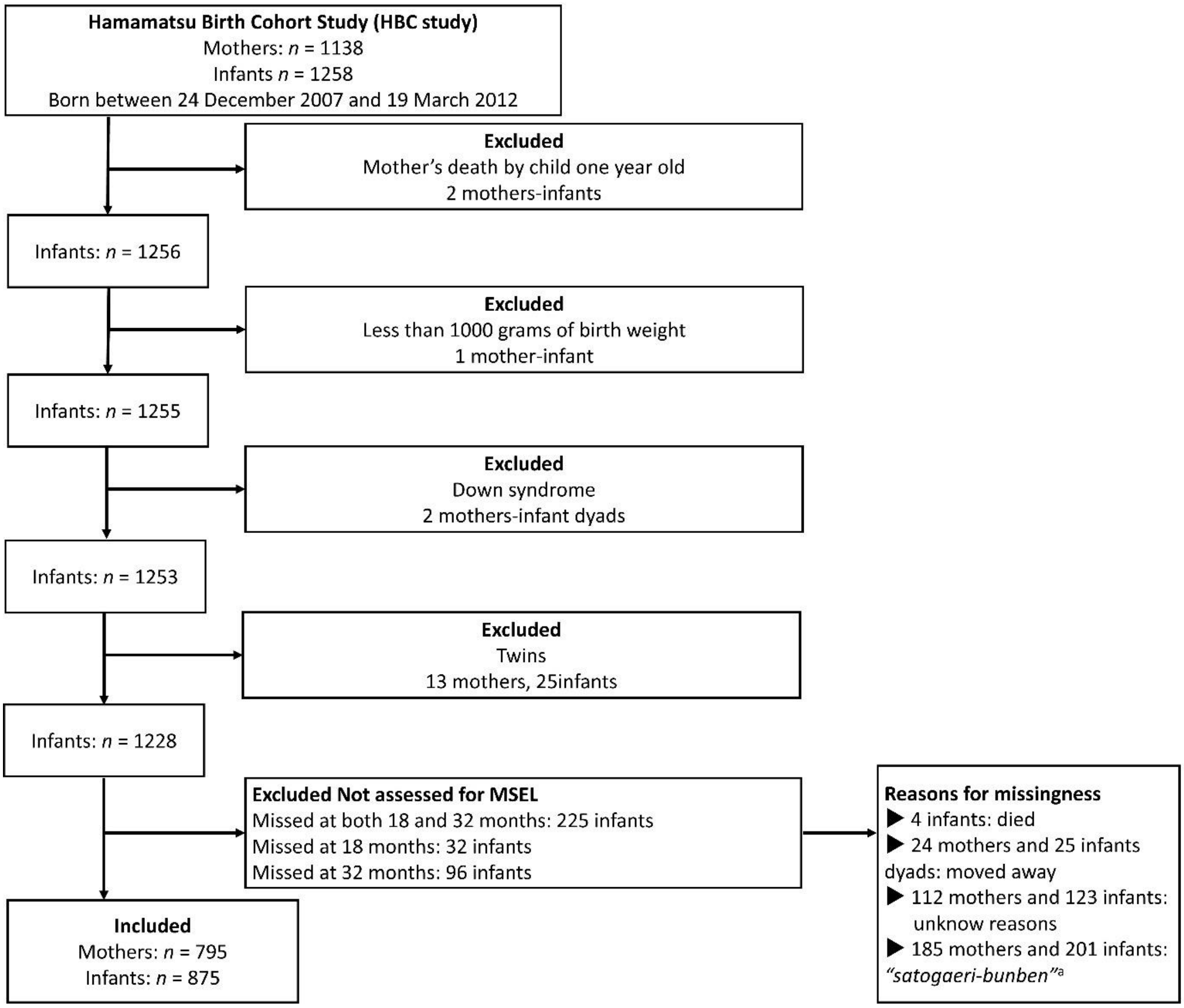

Supplementary Figure S1. **Participants flow chart**

[a] "Satogaeri-bunben" is a Japanese traditional support system for childbirth, in which some pregnant women return to their hometown for delivery while receiving family support.

MSEL, Mullen Scales of Early Learning.

Supplementary Table S1. Fit indices in latent class analysis

	Number of class						
	**1**	**2**	**3**	**4**	**5**	**6**	**7**
**Fit stats at time 1 (n = 875)**							
AIC	32873.9	32233.1	31981.6	31882.7	31802.6	31737.5	31714.6
BIC	32921.9	32309.9	32087.3	32017.2	31965.9	31929.5	31935.5
Entropy	-	0.856	0.786	0.739	0.786	0.807	0.806
Adjusted LMR-LRT p value	-	< 0.001	0.067	0.132	0.029	0.167	0.351
BLRT p value	-	< 0.001	< 0.001	< 0.001	< 0.001	< 0.001	< 0.001
	**Number of class**						
	**1**	**2**	**3**	**4**	**5**	**6**	**7**
**Fit stats at time 2 (n = 875)**							
AIC	32943.5	32079.2	31680.0	31577.7	31538.6	31489.7	31478.4
BIC	32991.5	32156.0	31785.6	31712.2	31701.9	31681.8	31699.4
Entropy	-	0.705	0.799	0.758	0.753	0.706	0.722
Adjusted LMR-LRT p value	-	< 0.001	< 0.001	0.003	0.037	0.054	0.353
BLRT p value	-	< 0.001	< 0.001	< 0.001	< 0.001	< 0.001	< 0.001

AIC, Akaike Information Criterion; BIC, Bayesian Information Criterion; BLRT, bootstrap likelihood ratio test; LMR-LRT, Lo-Mendell Rubin likelihood ratio test.

Supplementary Note 1. Details of the latent transition analysis in the present study

Latent transition analysis (LTA) enables us to identify the transition of classes assigned at different time-points and investigate the association of predictors (i.e., risk factors) with class shifts. The primary aim of LTA is to compute latent transition probabilities, which represent the likelihood that individuals change classes or remain in the same class across consecutive periods[1]. In the present study, we followed the three-step approach recommended for LTA[2,3]. In step 1, the LTA model without risk factors or covariates was estimated; in step 2, individuals were assigned to optimal latent transition patterns; in step 3, a model with risk factors or covariates was fit to detect relevant risk factors. We used this approach to identify transition class assignments, as well as to investigate risk factors for predicting transition patterns using Mplus, version 8[4]. However, during step 3, we failed to obtain accurate and reliable estimates due to the small number of assignment memberships in many of the created cells. In effect, fewer than nine children (less than 1%) were allocated to nearly half of the total 25 cells (12/25), which was created by 5 × 5 classes at time 1 and time 2 (see Table 2). Nine out of 12 belonged to descending or catching-up transition shifts, which were of interest in this study. Therefore, we modified the original step 3[3] and implemented the following process to enhance the precision of estimation and lead to clinically meaningful interpretations. First, we transferred the whole data on the individual latent class assignments at each time-point, transition assignments, and transition probabilities derived from Mplus to Stata software[5]. Then, we combined the three normal classes (i.e., High Normal, Normal, and Low Normal; see Table 2 and Fig. 1 in the main text) at each time-point into a single "3-Normals" class, resulting in reduction from five to three classes in total for both time 1 and time 2. Thus, the number of transition patterns was reduced from 25 to 9 (3 × 3) patterns. Second, we examined any differences in background characteristics across these three classes at time 1 to identify potential confounders (covariates) for subsequent analyses. Through the screening analytical steps, distributions of various factors such as sex, premature birth (<37 weeks), low placenta-to-birth weight ratio (<10th percentile), and household income were found to significantly differ across the three classes; thus, they were included as covariates in the analyses. Third, we used logistic regression analysis with the forward stepwise method in which the minimum number of risk factors associated with transition patterns was identified following the principle of parsimony, with covariates being compulsorily held to allow for their confounding effects. Notably, in the original three-step approach[3], risk factors and covariates are entered compulsorily, thereby rendering the derived estimates unreliable due to the inflated number of parameters. In this analysis, we regarded all the variables listed in Table 1 as risk factors, except for the four covariates. Here, we used the maximum class probability from LTA (converted into odds ratio) to account for variability in the likelihood of transition pattern assignments. Since two or more children born from the same mother were enrolled, such clustering was accounted for by using robust variance estimation. The results showed that maternal pre-pregnancy body mass index (>25 kg/m^2), maternal education, and infant's body mass index at 18 months (>1 standard deviation) were associated with descending transition patterns (see Table 3).

Supplementary Table S2. Association between covariates and neurodevelopmental transition patterns in the final model of multinomial logistic regression analysis (*n* = 811).

	Transition from 3-Normals at time 1 (765)		
	to 3-Normals at time 2 (681)	Delayed at time 2 (70)	Markedly Delayed at time 2 (14)
	Reference	OR [95% CI]	OR [95% CI]
**Covariates**			
Male sex	‥	1.51 [0.88, 2.60]	6.13 [1.44, 26.01]*
Premature birth before 37 weeks	‥	1.08 [0.33, 3.50]	5.12 [1.02, 25.63]*
Low placenta-to-birth-weight ratio (<10th percentile)	‥	1.48 [0.76, 2.87]	NA
Household income (million JPY)	‥	0.89 [0.78, 1.01]	0.93 [0.70, 1.23]
	**Transition from expressive language delayed at time 1 (19)**		
	to 3-Normals at time 2 (15)	Delayed at time 2 (3)	Markedly Delayed at time 2 (1)
	OR [95% CI]	OR [95% CI]	OR [95% CI]
**Covariates**			
Male sex	1.13 [0.40, 3.20]	NA	NA
Premature birth before 37 weeks	NA	0.90 [0.69, 1.18]	NA
Low placenta-to-birthweight ratio (<10th percentile)	NA	9.06 [0.54, 152.11]	NA
Household income (million JPY)	0.98 [0.81, 1.19]	0.90 [0.69, 1.18]	1.11 [1.01, 1.21]*
	**Transition from Delayed at time 1 (27)**		
	to 3-Normals at time 2 (9)	Delayed at time 2 (10)	Markedly Delayed at time 2 (8)
	OR [95%CI]	OR [95%CI]	OR [95%CI]
**Covariates**			
Male sex	2.73 [0.62, 11.96]	1.69 [0.42, 6.68]	NA
Premature birth before 37 weeks	2.40 [0.41, 13.82]	3.18 [0.27, 37.10]	NA
Low placenta-to-birthweight ratio (<10th percentile) [a]	1.38 [0.20, 9.39]	1.86 [0.39, 8.80]	2.70 [0.59, 12.31]
Household income (million JPY)	0.61 [0.47, 0.79]****	0.73 [0.51, 1.03]	0.99 [0.77, 1.26]

*P < .05. **P <.01. ***P <.005. ****P <.001.
OR, odds ratio; CI, confidence interval; JPY, Japanese Yen; NA, not available.

Supplementary Note 2. Results and inferences of covariates for neurodevelopmental transition patterns.

The variables of male sex (OR = 6.13; 95% CI: 1.44, 26.01) and premature birth before 37 weeks (OR = 5.12; 95% CI: 1.02, 25.63) were significantly associated with the "3-Normals to M-Delayed" descending transition pattern. These variables were treated as covariates to adjust for background characteristic differences across three classes at time 1 so that they could not be considered as risk factors. Nevertheless, given that any existing confounding and risk-increasing effects in a single variable cannot be separated, there is the possibility that these two factors may in effect have contributed to the descending transition. Compatible with this inference, a previous study has reported that male sex and prematurity were associated with a diversion from normal to delayed development in the neurocognitive domain during age intervals similar to our study[6].

Household income used as a covariate was negatively associated with the catching-up transition pattern from Delayed to 3-Normals; infants from *low* income were *more* likely to make the catching-up transition from the Delayed class at time 1 to the 3-Normals class at time 2, with the odds ratio (OR) being 0.61 (95% CI: 0.47, 0.79) when compared with infants who transitioned from 3-Normals at time 1 to 3-Normals at time 2. Given that higher income has been reported to be positively related to child development[7], our contradictory finding is puzzling. One possible interpretation is an involvement of public health care professionals in supporting the development of infants with socioeconomically deprived backgrounds[7,8]. Those infants from low socioeconomic status identified as the Delayed class at time 1 in this study may have naturally raised more attention from health care providers (e.g., through nationwide infant check-ups)[9] and prompted intervention to foster their development.

Supplementary Table S3. Comparison of participants' characteristics between the group included in the analysis and the group excluded from the analysis

	Included (n = 875)	Excluded (n = 383)	
	**Mean (SD)**	**Mean (SD)**	***P* value[a]**
**Infants**			
Birth weight (g)	2957.0 (422.2)	2902.4 (472.8)	0.042
Gestational age at birth (weeks)	39.0 (1.4)	38.6 (1.8)	< 0.0001
	**n (%)**	**n (%)**	***P* value[a]**
Sex			
Male	432 (49.4%)	216 (56.4%)	0.022
Female	443 (50.6%)	167 (43.6%)	
Prematurity			
<37 weeks	46 (5.3%)	40 (10.4%)	0.0011
≥37 weeks	829 (94.7%)	343 (89.6%)	
Small for gestational age			
<10th percentile	77 (8.8%)	29 (7.6%)	0.47
10th–100th percentile	798 (91.2%)	354 (92.4%)	
Age-standardised BMI scores at 18 months			
>1 standard deviation	50 (6.1%)	3 (2.5%)	0.11
<1 standard deviation	776 (93.9%)	116 (97.5%)	
	**n (%)**	**n (%)**	***P* value[a]**
**Parents**			
Maternal BMI at pre-pregnancy (kg/m^2)			
>25.0	100 (11.4%)	30 (7.8%)	0.054
<25.0	775 (88.6%)	353 (92.2%)	
Placenta-to-birth-weight ratio (twin excluded)			
<10th percentile	152 (17.7%)	65 (19.5%)	0.46
10th–100th percentile	707 (82.3%)	268 (80.5%)	
	**Mean (SD)**	**Mean (SD)**	***P* value[a]**
Paternal age at birth (yr)	33.5 (5.7)	32.5 (6.0)	0.0065
Maternal age at birth (yr)	31.7 (5.0)	30.7 (5.1)	0.0005
Paternal education (yr)	14.1 (2.6)	13.8 (2.8)	0.059
Maternal education (yr)	13.8 (1.8)	13.6 (2.2)	0.028
Household income (million JPY)	6.0 (2.7)	5.9 (2.9)	0.41

SD, standard deviation; BMI, body mass index; JPY, Japanese Yen.
[a] *t*-test for continuous variables and the chi-square test for categorical variables were used.

Supplementary Table S4. Association between possible risk factors and neurodevelopmental transition patterns in the final model of multinomial logistic regression after excluding infants with a clinical diagnosis of autism spectrum disorder (n = 26)

	Transition from 3-Normals at time 1 (746)		
	to 3-Normals at time 2 (673)	Delayed at time 2 (62)	Markedly Delayed at time 2 (11)
	Reference	OR [95%CI]	OR [95%CI]
**Possible risk factors**			
Maternal overweight status at pre-pregnancy	..	2.61 [1.25, 5.43]**	NA
Low maternal education (yr)	..	1.23 [1.06, 1.42]	1.08 [0.78, 1.49]
Male sex [a]	..	1.41 [0.79, 2.51]	10.96 [2.47, 48.51]***
Small for gestational age	..	2.07 [0.82, 5.22]	0.68 [0.056, 8.50]
Premature birth before 37 weeks [a]	..	1.16 [0.36, 3.81]	8.69 [1.74, 43.39]**
Infant overweight status at 18 months	..	1.65 [0.62, 4.34]	8.8 2[1.88, 41.36]**
Low placenta-to-birthweight ratio (<10th percentile) [a]	..	1.52 [0.74, 3.12]	NA
Household income (million JPY) [a]	..	0.90 [0.79, 1.01]	0.91 [0.66, 1.26]

	Transition from Expressive Language Delayed at time 1 (17)		
	to 3-Normals at time 2 (15)	Delayed at time 2 (2)	Markedly Delayed at time 2 (0)
	OR [95%CI]	OR [95%CI]	OR [95%CI]
**Possible risk factors**			
Maternal overweight status at pre-pregnancy	0.90 [0.11, 7.31]	NA	NA
Low maternal education (yr)	1.23 [1.01, 1.49]*	3.12 [0.59, 16.66]	NA
Male sex [a]	1.17 [0.41, 3.33]	NA	NA
Small for gestational age	0.92 [0.11, 7.45]	33.23 [0.27, 3845.80]	NA
Premature birth before 37 weeks [a]	NA	140.01 [0.0094, 2 0652.31]	NA
Infant overweight status at 18 months	NA	NA	NA

Low placenta-to-birthweight ratio (<10th percentile) [a]	2.90 [0.94, 8.94]	NA	NA
Household income (million JPY) [a]	0.99 [0.82, 8.94]	1.16 [0.92, 1.46]	NA
	**Transition from Delayed at time 1 (22)**		
	to 3-Normals at time 2 (8)	Delayed at time 2 (10)	Markedly Delayed at time 2 (4)
	OR [95%CI]	OR [95%CI]	OR [95%CI]
**Possible risk factors**			
Maternal overweight status at pre-pregnancy	5.68 [1.08, 29.77]*	0.87 [0.088, 8.52]	NA
Low maternal education (yr)	1.05 [0.68, 1.63]	1.28 [0.84, 1.92]	2.01 [1.08, 3.70]*
Male sex [a]	2.21 [0.48, 10.02]	1.74 [0.43, 6.93]	NA
Small for gestational age	11.86 [2.42, 58.12]***	NA	NA
Premature birth before 37 weeks [a]	2.65 [0.44, 15.75]	3.15 [0.26, 37.25]	NA
Infant overweight status at 18 months	3.19 [0.24, 40.98]	2.10 [0.23, 18.94]	NA
Low placenta-to-birthweight ratio (<10th percentile) [a]	1.58 [0.22, 11.17]	1.88 [0.40, 8.77]	2.96 [0.38, 22.89]
Household income (million JPY) [a]	0.66 [0.54, 0.81]****	0.74 [0.53, 1.04]	1.11 [0.86, 1.43]

OR, odds ratio; CI, confidence interval; JPY, Japanese Yen; NA, not available.
[a] included as covariates.
**P* <.05. ***P* <.01. ****P* <.005. *****P* <.001.

Supplementary Table S5. Association between neurodevelopmental transition patterns and adaptive behaviour at 40 months in linear regression analysis[a] after excluding infants with a clinical diagnosis of autism spectrum disorder (n=26)

	**Adaptive Behaviour Composite standardised score** coefficient (95%CI)
**Transition patterns (n)**	
3-Normals to 3-Normals (646)	reference
3-Normals to Delayed (59)	-7.52 [-9.41, -5.62]****
3-Normals to Markedly Delayed (10)	-12.05 [-18.71, -5.40]****
EL-Delayed to 3-Normals (14)	-2.44 [-5.24, 0.34]
Delayed to 3-Normals (8)	0.47 [-4.44, 5.39]
EL-Delayed to Delayed (2)	-15.24 [-17.37, -13.11]****
EL-Delayed to Markedly Delayed (0)	..
Delayed to Delayed (10)	-9.85 [-13.47, -6.23]****
Delayed to Markedly Delayed (4)	-18.61 [-24.01, -13.22]****

EL-Delayed, Expressive Language Delayed.

[a] Covariates used in this analysis comprised infant's sex (male), premature birth (<37 weeks), low placenta-to-birthweight ratio (<10th percentile), household income, maternal body mass index at pre-pregnancy (>25 kg/m^2), small for gestational age (<10th percentile), infant’s standardised body mass index at 18 months of age (>1 SD), and maternal educational history.

****P <.001.

References

1 Collins, L. M. & Lanza, S. T. *Latent class and latent transition analysis: With applications in the social, behavioral, and health sciences*. Vol. 718 (John Wiley & Sons, 2010).

2 Muthen, B. & Muthen, L. K. Integrating person-centered and variable-centered analyses: growth mixture modeling with latent trajectory classes. *Alcohol. Clin. Exp. Res.* **24**, 882-891 (2000).

3 Nylund-Gibson, K., Grimm, R., Quirk, M. & Furlong, M. A latent transition mixture model using the three-step specification. *Struct. Equ. Modeling.* **21**, 439-454 (2014).

4 Muthén, L. K. & Muthén, B. O. *1998–2012. Mplus user's guide*. (Muthén & Muthén, 2012).

5 StataCorp, L. *Stata treatment-effects reference manual*. (A Stata Press Publication, 2015).

6 Hillemeier, M. M., Morgan, P. L., Farkas, G. & Maczuga, S. A. Perinatal and socioeconomic risk factors for variable and persistent cognitive delay at 24 and 48 months of age in a national sample. *Maternal and Child Health Journal* **15**, 1001-1010 (2011).

7 Demirci, A. & Kartal, M. Sociocultural risk factors for developmental delay in children aged 3–60 months: a nested case-control study. *Eur. J. Pediatr.* **177**, 691-697 (2018).

8 Ozkan, M., Senel, S., Arslan, E. A. & Karacan, C. D. The socioeconomic and biological risk factors for developmental delay in early childhood. *Eur. J. Pediatr.* **171**, 1815-1821 (2012).

9 Kamio, Y. *et al.* Brief Report: Best Discriminators for Identifying Children with Autism Spectrum Disorder at an 18-Month Health Check-Up in Japan. *J. Autism Dev. Disord.* **45**, 4147-4153 (2015).
